# CCS-DTN: Clustering and Network Coding-Based Efficient Routing in Social DTNs

**DOI:** 10.3390/s150100285

**Published:** 2014-12-25

**Authors:** Zhenjing Zhang, Maode Ma, Zhigang Jin

**Affiliations:** 1 Tianjin University, Tianjin 300072, China; E-Mail: zgjin@tju.edu.cn; 2 Nanyang Technological University, Singapore; E-Mail: EMDMa@ntu.edu.sg

**Keywords:** delay tolerant network, routing, clustering, social DTN, delivery probability

## Abstract

With the development of mobile Internet, wireless communication via mobile devices has become a hot research topic, which is typically in the form of Delay Tolerant Networks (DTNs). One critical issue in the development of DTNs is routing. Although there is a lot research work addressing routing issues in DTNs, they cannot produce an advanced solution to the comprehensive challenges since only one or two aspects (nodes' movements, clustering, centricity and so on) are considered when the routing problem is handled. In view of these defects in the existing works, we propose a novel solution to address the routing issue in social DTNs. By this solution, mobile nodes are divided into different clusters. The scheme, Spray and Wait, is used for the intra-cluster communication while a new forwarding mechanism is designed for the inter-cluster version. In our solution, the characteristics of nodes and the relation between nodes are fully considered. The simulation results show that our proposed scheme can significantly improve the performance of the routing scheme in social DTNs.

## 1. Introduction

A Delay Tolerant Network (DTN) presents a new communication model, which due to the nature of the network uses a store-carry-forward approach to deliver messages. One of its main characteristics is that the links between mobile nodes are volatile and may break down for a long period of time at any time. With the development of the mobile Internet, research on DTNs has become a hot research topic. There are many ubiquitous scenarios similar to the communication ways of DTNs, including social networks. In this paper, we mainly focus on the DTNs with social characteristics, called social DTNs. In social DTNs, people with some social relationship tend to be co-located quite regularly and people with same characteristics, including interests, nationality, age, *etc.*, usually appear in the same place. As a result, communities will be established by the people who collaborate with each other frequently. In each community, people can be considered as “friends” and messages among them can be efficiently delivered. What's more, there are several active persons in the social DTNs who move frequently in different communities and come into contact with many other peoples. These active persons can take on the responsibility of transmitting messages between communities.

Considering the characteristics of social DTNs, there are several issues that need to be solved. They include: (1) the reduction of messages dropped due to the unreliability of the wireless communication links; (2) the need for a mobility model which can accurately reflect real scenarios; (3) the need for a mechanism which can make full use of the social features of the mobile nodes and the relationships among them. The existing research solutions have not solved these problems efficiently, as will be introduced in the related works. In this paper, we propose a new routing scheme to solve these issues, named CCS-DTN. With CCS-DTN, firstly a mobility model is proposed to describe the social DTN scenarios. Secondly, according to nodes' contact probability, mobile nodes are divided into different clusters. Then different routing mechanisms are designed for intra-cluster and inter-cluster routing. The Spray and Wait scheme is used for the intra-cluster routing while a new forwarding mechanism is proposed for the inter-cluster routing problem. In the proposed mechanism, messages are forwarded to active nodes and then these active nodes adopt the “carry-forward” method to transmit messages. Finally, to solve the problem of messages dropped due to unreliability of the wireless communication channels, the messages will be coded by the random linear network coding scheme for multiple transmissions.

Our major contributions presented in this paper include: (1) a mobility model which can accurately describe real scenarios; (2) a dynamic clustering mechanism which highlights the community characteristics of the social DTNs; (3) a new routing mechanism which takes full advantages of the characteristics of the active nodes; (4) the network coding mechanism employed to reduce the negative effects caused by the unreliability of the wireless communication channels.

The remainder of this paper is organized as follows: in Section 2, the related works is introduced. The mobility model and the proposed routing mechanism are presented in Section 3. In Section 4, the performance of the solution is evaluated by a mathematical model and simulation experiments. Finally, the conclusion of the paper is summarized in Section 5.

## 2. Related Works

A lot of research works have been done on efficient routing in a DTN environment. According to the main technology used in the routing, we classify the current research works as follows: routing based on history information, routing based on clustering, routing based on network coding and routing based on social networks.

### 2.1. Routing Based on History Information

Pioneering studies on routing in DTNs have mainly focused on the actions required for the next hop transmission with consideration of historic information. Typical protocols include Epidemic [1], Prioritized Epidemic (PREP) [2], Spray and Wait [3], Seek and Focus [4] and Probabilistic Routing Protocol using History of Encounters and Transitivity (PROPHET) [5] and some other improvements and variations have been proposed. By the Epidemic scheme, a node with messages will forward a copy of each message to any node it meets and the messages are distributed transitively through the networks. Through such transitive transmission, the messages have a higher probability to reach their destinations. Based on the Epidemic scheme, the PREP scheme prioritizes the messages based on the costs to the destination, the source and the expiring time. Different from the Epidemic and the PREP schemes, the Spray and Wait routing approach limits the number of message copies and works in two phases, namely the Spray phase and the Wait phase. For each message originating at a source node, *L* message copies are initially spread to *L* distinct “relays” and then the *L* relays will forward each message only to its destination. By the Seek and Focus scheme, local forwarding decisions are made based on the current connectivity and the information of the predictions of future connectivity for a single copy of each message. The PROPHET scheme is a routing mechanism working based on the history information, which is the history of the encounters and transitivity to select next hop for the message forwarding. The transmit predictability *P*_(_*_a_*_,_*_b_*_)_ ∈ (0,1] has been adopted as the probability metrics. The main characteristic of the PROPHET scheme is that node *i* will forward a message to node *j* if node *j* has a higher predicted probability to the destination of the message than that of node *i*.

### 2.2. Routing Based on Clustering

Although the protocols with history information work well enough to be able to achieve high message delivery ratios, messages are delivered with a high latency. To reduce the delay, other properties such as mobility and the relationship among mobile nodes have been considered to make routing decisions. In some particular DTN environments, clustering with hierarchical structures has been proposed to reduce the end-to-end delay. In [6], a hierarchical forwarding mechanism has been proposed to group the nodes according to their encounter frequency. Initially, each node is considered as a cluster consisting of a single node. And the operation which combines the two best clusters to form a new cluster, determined by the distance function, is repeated until the cluster with all nodes is finally formed. Similar works using clustering technology can be found in [7–9]. In [7], the maximum diameter is used for the clustering and link similarity has been adopted in [9]. In [8], similar mobility pattern is used for the clustering and a new cluster-based routing protocol has been proposed. By the proposed protocol, different routing schemes have been adopted for the intra-cluster and the inter-cluster routing. For the intra-cluster routing, direct transmission is used while gateway nodes are used to exchange messages for the inter-cluster routing. By these protocols, the mobile nodes with frequent contact and the mobile nodes with less contact will be differently treated, thus improving the message transmission efficiency. However, the messages' destinations may not be able to receive all the messages due to the unreliability of the wireless communication channels.

### 2.3. Routing Based on Network Coding

It is clear that the transmission reliability could be improved when a network coding technique is used. Some existing researches show that applying network coding techniques to DTN environments can improve the performance in terms of the message loss rate. In [10], an efficient network coding scheme for DTN has been proposed to analyze the redundancy of the coded messages which has significant advantages in enhancing the message delivery ratio and reducing the transmission overhead. In [11], based on the fact that there are some mobile nodes, denoted as HUBs, which have frequent contacts with other mobile nodes, a new mechanism, Message Forwarding using Hub-based Network (HUBCODE), has been proposed to use the random linear network coding scheme to address the routing issue, which can obtain 20% improvement.

### 2.4. Routing Based on Social Networks

By the abovementioned routing schemes, good performance may not be obtained in some DTNs which have the characteristics of social networks and it has been proven in the following references. Existing studies show that in such scenarios, there are some active nodes which can transmit messages to their destinations with less hops. For example, in a campus scenario, students in the same group contact frequently while students in different groups have less contact, but a group leader has more contacts between different groups. Some new schemes have been proposed to address routing issues in such DTNs. In [12] the application of these characteristics to communication systems have been highlighted. In [13], the authors have derived the optimization of the routing policy in such social DTNs. In [14], based on the small world theory, a routing algorithm has been proposed to combine the concepts of similarity and centrality, where the similarity refers to the number of the same neighbors of two nodes while the ratio of the number of the shortest paths including a node over the number of all the shortest paths is defined as the centrality of the node. Additionally, in [15] in accordance with the two important characteristics of a social network, community and centrality, the authors have proposed a forwarding algorithm, by which messages will be constantly forwarded to the nodes with the higher centrality since these nodes will have higher probability to meet the destination node of the messages. Similar works can also be found in [16,17]. More social characteristics such as the social distance defined in [18], the asynchronous centrality defined in [19], the social links of the nodes pairs defined in [20], the node's social relation defined in [21], the impact of strangers defined in [22] and the group movement defined in [23] can be employed to make the forwarding decision. A more efficient combination of the social features of the mobile nodes and the relationship among them is expected to achieve much better performance.

## 3. The Proposed Routing Mechanism

The notations used in the scheme are shown in Table 1.

### 3.1. Mobility Model

**Figure 1.**
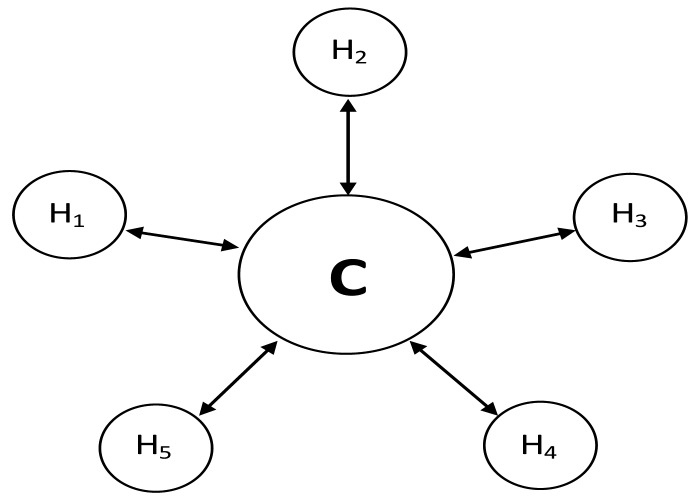
Mobility Model.

In this paper, the system under the study is a social DTN, where each mobile node is in continuous movement. To simplify the problem, we have adopted the mobility model described in [8] with some modifications, because the model can reflect the characteristics of the scenarios (campus, international conference) which have been investigated in this paper. The model in [8] can accurately reflect the characteristics of the scenarios but it has not made full use of the characteristics of the nodes. According to the analysis in [15], there are approximately 20% of the nodes which have extremely high relaying ability. That is to say, these 20% nodes are more active than other nodes. Therefore, to accurately describe the characteristics of the nodes, 20% of the nodes have been set to be active than other nodes in the modified mobility model. In the system, there are five hot spots and one cold spot denoted as *H*_1_ − *H*_5_ and *C* as shown in Figure 1. Each mobile node is assumed to have a “home” hot spot where it stays most of its time but not all the hot spots. And each mobile node always goes to the cold spot when it leaves a hot spot. For any node *a* (assume it belongs to the hot spot *H_1_*), we assume when it leaves *H_1_*, it always goes to the cold spot. The assumption is to make the model closer to the reality. For example, in a campus scenario (one of scenarios in social DTNs), a hot spot may be a department where students stay together, while a cold spot would correspond to the transition between two hot spots. In other words, before entering other hot spots, node *a* needs have a transition process when it leaves *H_1_*. It is further assumed that 20% of the nodes will take more time to move among different spots than other nodes.

**Figure 2.**
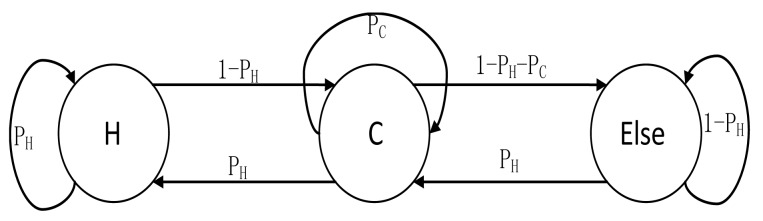
Change of the State of a Node.

As shown in Figure 2, when a node is home, it will have a probability of *P_H_* to stay or 1 − *P_H_* to move to the cold spot in the next time slot. While at the cold spot, it will go home with probability of *P_H_*, or have a probability of *P_C_* to stay, or move to other hot spot with probability of 1 − *P_H_* − *P_C_*, where (*P_H_*+*P_C_* < 1).Finally, when the node stays at a hot spot which is not its home, it will have a probability of *P_H_* to move to the cold spot or stay with probability of *1* − *P_H_*.

The WiFi technology has been adopted for the communication among these nodes. Each node will have a unique ID and maintain the contact information with others by a list of parameters including node ID, contact probability, and contact time. The parameter of the contact probability is automatically updated in each time slot according to the contact time.

Based on the mobility model, some communities can be formed from time to time in the system and nodes within the same community contact frequently with each other. The message forwarding inside one community is simple. On the other hand, mobile nodes in different communities have little contact but there exist some active nodes moving among communities which can be used to transmit messages from one community to another as relay nodes. As a result, clustering technology can be adopted in our new routing protocol, CCS-DTN. And different forwarding mechanisms could be used for the intra-cluster and the inter-cluster routing. By the clustering technique, each node will belong to a cluster with a cluster ID and maintain the information of the members in the cluster by a list of parameters such as the node ID. The CCS-DTN protocol consists of two phases, which are the phase of cluster operation and the phase of message delivery. Since the devices in social DTNs, such as mobile phone, are smart and have time information, it can be assumed that the network is a synchronized network.

### 3.2. Functions and Parameters

*Definition 1: Contact Probability*: The Contact Probability describes that how likely it is for two nodes to communicate in a time slot. Node *i* maintains an entry of contact probability *ε_ij_* for node *j*, which is updated in each time slot according to the formula (1) as follows. That is to say, if node *i* meets node j in a time slot, *ε_ij_* will be updated as (1 − *α*)[*ε_ij_*] + *α*. Otherwise, it will be updated as (1 − *α*)[*ε_ij_*]. When two nodes meet at the first time, there will be an initial value of the contact probability for these two nodes. The term *α* is a constant value which will be evaluated according to different scenarios:

(1) $${ɛ}_{ij}=\{\begin{array}{c} \left(1-\alpha \right)\left[{ɛ}_{ij}\right]+\alpha i\text{meets}j\\ \left(1-\alpha \right)\left[{ɛ}_{ij}\right]\text{otherwise} \end{array}$$

Obviously, when nodes meet frequently, the probability will increase over time. Otherwise, it will become lower. In this way, we can predict the probability of future contact for different nodes, which provides the basis for the message forwarding.

*Definition 2. Node's Stability*: The Node's Stability describes how likely it is a node will meet any other nodes in the same cluster. A node with a higher stability will have a higher chance to meet any other nodes in the same cluster and then to forward a message. The stability of node *i* is the minimum contact probability between node *i* and the other nodes in *C_i_*, which is denoted *S_i_* and is calculated as follows:

(2) $$S_{i}=\text{min}\left\{{ɛ}_{ik}|k\in C_{i}\right\}$$

*Definition 3. Nodes' Synchronization*: If node *i* and node *j* need synchronization, node *i* sends node *j* a list of its cluster members. Upon receiving the list, node *j* divides it into two subsets according to the contact time:

(3) $$\Psi_{1}=\left\{k|T_{i}^{k}>T_{j}^{k},k\in M_{i},k\notin M_{j}\right\}$$

(4) $$\Psi_{2}=\left\{k|T_{i}^{k}\leq T_{j}^{k},k\in M_{i},k\notin M_{j}\right\}$$

where, *Ψ*_1_ is a set including a list of nodes in which node *i* has the latest update that is not known by node *j*. Similarly, in *Ψ*_2_, node *j* has the latest update which is not known by node *i*. As a result, node *j* updates the list of the members in *M_j_* by adding the node in *Ψ*_1_. Meanwhile, node *j* sends *Ψ*_2_ to node *i*, which updates the list of the members in *M_i_* by removing the node in *Ψ*_2_. Next, node *j* sends node *i* the list of its cluster members for a similar process.

*Definition 4. Node's Centrality*: The centrality of node *i* is the number of different clusters that the node *i* meets per unit time. Node *i*'s centrality, denoted as *CT_i_*.

*Definition 5. Random Linear Network Coding*: Suppose the source node generates *n* messages *p*_1_, *p*_2_, …*p_n_* with the same destination. By using the coding method, $c_{x}\left(1\leq x\leq m\right)=\sum_{k=1}^{n}e_{k}^{x}p_{k}$, we can obtain *m* (*m*>*n*) coded messages *c*_1_, *c*_2_, …*c_m_*. To be noted that $e_{k}^{x}\left(1\leq x\leq m,1\leq k\leq n\right)$ *i*s randomly generated from a finite field and each arithmetic is carried out in the finite field. And then each coded message *c_x_*(1 ≤ *x* ≤ *m*) will be forwarded with the vector $\left[e_{1}^{x},e_{2}^{x},\ldots e_{n}^{x}\right]$. When the destination receives *n* coded messages *c*_1_, *c*_2_, …*c_n_*, which are linearly independent, it can decode the original messages *p*_1_, *p*_2_, …*p_n_*.

### 3.3. Clustering Algorithm

According to the stability of the node, the clustering algorithm determines whether a node joins or leaves a cluster. The algorithm is event-driven. Two possible events could happen at each node, which are Slot-Timeout and Meet-A-Node. The Slot-Timeout event is when at the end of each timeslot, the contact probability lists at each node will be updated according to the definition of contact probability (Definition 1). The Meet-A-Node event describes that when two nodes come to communication, they will exchange and update their clustering information.

As shown in Algorithm.1, at the end of each time slot, the contact probability will be updated according to formula (1). When node *i* meets node *j*, according to whether node *i* and node *j* belong to the same cluster, there are two cases.

If node *i* and node *j* are in the same cluster, it needs to verify whether the two nodes still stay in the same cluster. If the contact probability between node *i* and node *j* is lower than or equal to the given threshold *γ*, one of them has to leave the cluster. First, we compute the stability of node *i* in the cluster which do not include node *j* and the stability of node *j* in the cluster which do not include node *i*. The node with lower stability will leave the cluster and form a new cluster containing itself. Otherwise, synchronization between node *i* and node *j* is required.


**Algorithm 1**: the clustering phase of the proposed CCS-DTN
**INPUT: nodes with contact probability list, stability****OUTPUT: different clusters**
**Upon** the Slot-Timeout Event **do**updateContactProbability(); //according to (1)**Upon** the Meet-A-Node Event **do** //for any two node (node *i, j*)
**if**
*C_i_* = = *C_j_***then****if**
*C_i_* ≠ *C_j_***then** **if**
*ε_ij_* ≤ *γ*
**then** **if** ∃*k* ∈ *C_j_, ε_ik_* ≤ *γ* **if** (*S_i_* ≤ *S_j_*) **then** &&∃*k* ∈ *C_i_, ε_jk_* ≤ *γ*
**then** removeNode(*i, C_j_*); noAction(); **else** **else if** ∃*k* ∈ *C_j_, ε_ik_* ≤ *γ*
**then** removeNode(*j, C_i_*); addNode(*i, C_j_*); **else** **else if** ∃*k* ∈ *C_i_, ε_jk_* ≤ *γ*
**then** nodeSynchronization(*i, j*); addNode(*j, C_i_*);**end** **else** **if**
*S_i_* ≤ *S_j_*
**then** addNode(*i, C_j_*); **else** addNode(*j, C_i_*);**end**


**Figure 3.**
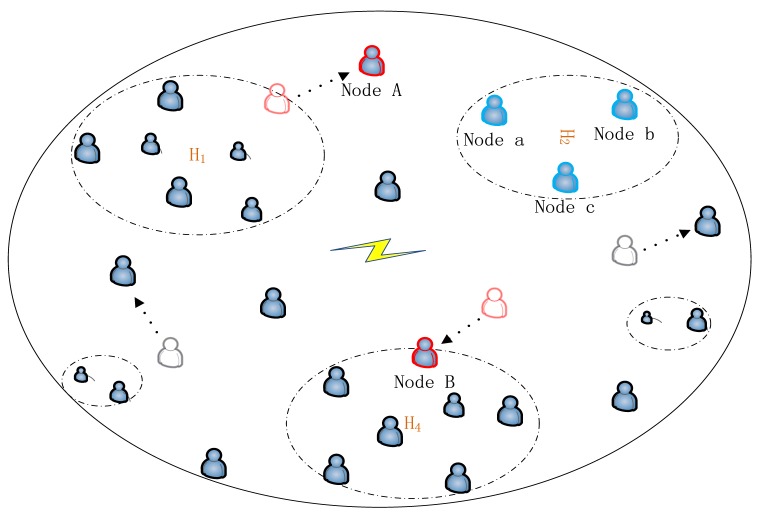
Clustering Process.

On the other hand, when node *i* and node *j* are in different clusters, a verification function will be invoked to check whether the two nodes need to update their clusters. Specifically, a node *i* can be added to a cluster *C_j_* if the contact probability between node *i* and each node in *C_j_* are greater than the given threshold *γ*. As shown in Figure 3, there are four cases to be processed. (1) If node *i* cannot join *C_j_* and node *j* cannot join *C_i_*, no action is required; (2) If node *i* can be added to *C_j_* and node *j* cannot be added to *C_i_*, add node *i* to *C_i_* and update *M_i_, M_j_*; (3) If node *i* cannot be added to *C_j_* and node *j* can be added to *C_i_*, add node *j* to *C_i_* and update *M_i_, M_j_*; (4) If node *i* can join *C_j_* and node *j* can join *C_i_*, add the node which has lower stability to the other node's cluster and update *M_i_, M_j_.*

According to the clustering mechanism, there are three situations which are shown in Figure 3: (1) in a hot spot, nodes contact frequently with each other and they will be grouped into one cluster, such as node *a*, node *b* and node *c*; (2) in the movement, a node maybe leave it's hot spot, such as node *A*. In this situation, according the clustering mechanism, node *A* will leave its original cluster and form a new cluster including itself; (3) in contrast, a node maybe join a hot spot, such as node *B*. it will join the cluster in *H_3_*.

### 3.4. CCS-DTN Routing

After clustering, every node will belong to a cluster. As shown in Algorithm 2, it is assumed that if node *i* needs to send messages to node *j*, there could be only two cases, which are that the two nodes are in the same cluster and that the two nodes belong to different clusters. And when messages are transmitted over clusters, the network coding technique will be used.


**Algorithm 2**: Routing Phase of the Proposed CCS-DTN
**INPUT: messages, any node *i*****OUTPUT: messages are forwarded to the destinations**
**Upon** reception of message *m*
**do****Upon**
*i* meets *j*
**do**
**if**
*i* is the destination node *d* of *m*
**then****if**
*C_i_* == *C_j_*
**then** messageDelivered(*m*); sprayAndWait(*i,j*);**if**
*i* is the source node *s* of *m*
**then****else** **if**
*C_i_* == *C_d_*
**then** **foreach** message *m* in node *i*
**do** cacheIntraClusterMessage(*m*); **if**
*C_j_* == *C_d_*
**then** **else** messageAllTrans(*m,i,j*); cacheInterClusterMessage(*m*); **else** checkForCoding(); computeCT(*i,j*);**if**
*i* is a relay node **then** **if**
*CT_i_* < *CT_γ_* &&*CT_j_* ≥ *CT_γ_*
**then** **if**
*m* is an intra-cluster message **then**  
$nm=\frac{CT_{j}}{CT_{i}+CT_{j}}$ of the copies cacheIntraClusterMessage(*m*); **else** messagePartTrans(*m,i,j,nm*); **if**
*C_i_* == *C_d_*
**then** **if**
*CT_i_* ≥ *CT_γ_* &&*CT_j_* ≥ *CT_γ_*
**then** cacheIntraClusterMessage(*m*); *nm* = half of the copies **else** messagePartTrans(*m,i,j,nm*); cacheInterClusterMessage(*m*); **foreach** message *m* in node *j*
**do****end** //the transmission is same as that of node *i***end**


#### 3.4.1. Intra-Cluster Routing

According to the clustering algorithm, the contact probability between nodes in the same cluster is greater than the given threshold, so we can adopt the routing scheme which limits the number of copies of a message. Spray and Wait with two copies of a message can be adopted as the intra-cluster routing scheme in the proposed solution. Since within the same cluster, nodes have high contact probability with each other, two copies for Spray and Wait are enough to obtain a high delivery rate.

#### 3.4.2. Inter-Cluster Routing

**Figure 4.**
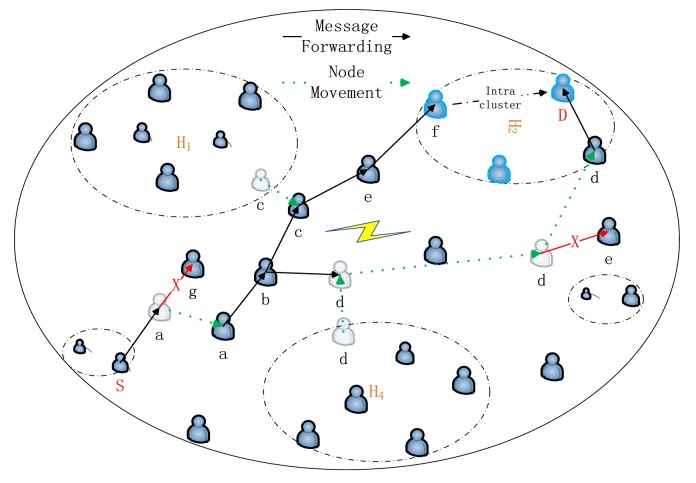
The process of inter-cluster routing.

A new forwarding mechanism has been proposed for inter-cluster routing. By the new mechanism, the parameter of centrality is used to determine the next hop node. When the source node and the destination node do not belong to the same cluster, the source node will first accumulate and encode a sufficient number of messages. Then the coded messages will be forwarded to the relay nodes with a higher centrality. With the movement of the relay nodes, messages will be gradually delivered to the destinations or the nodes in the same cluster with the destination node. When messages are forwarded to the node which belongs to the same cluster with the destination node, they can be delivered to the destination using intra-cluster routing. For example, in Figure 4 we assume *CT_s_* < *CT_a_* < *CT_b_* < *CT_c_* < *CT_e_* < *CT_f_, CT_a_* > *CT_g_, CT_b_* > *CT_d_, CT_d_* > *CT_e_*. From Figure 4, the source node *S* has messages to be transmitted to the destination node *D*. Since node *S* and node *D* are not in a same cluster, the inter-clustering routing is used. Firstly, when messages are cached in *S*, they will be coded using a random linear network coding scheme. Secondly, the coded messages will be forwarded according to the node's centrality. From Figure 4, messages will be forwarded to the destination by two ways. In the way of *S*->*a*->*b*->*d*->*D*, messages are directly transmitted to the destination *D* while in the way of *S*->*a*->*b*->*c*->*e*->*f*->*D*, messages are transmitted to node *f* first and then the messages are delivered from node *f* to node *D* using intra-cluster routing.

By the forwarding mechanism messages are accumulated at the nodes with higher centrality. These nodes may suffer from buffer overflow, since the buffer size at each node is limited. In this way, the delivery ratio will be reduced. Therefore, a load balancing mechanism is required to handle it. First, a threshold *CT_γ_* is pre-set. If node *i* with centrality less than *CT_γ_* meets node *j* with a higher centrality, node *i* will send *CT_j_*/(*CT_i_* + *CT_j_*) numbers of the copies to node *j*. In this way, messages will be forwarded to multiple nodes with higher centrality, thus avoiding the risk of the messages to be lost at one node. And the node with a higher centrality will have more messages. If node *i* with a centrality greater than that of *CT_γ_* meets node *j* with a greater centrality than that of *CT_γ_*, node *i* will send half number of the copies to node *j*.

## 4. Performance Analysis

### 4.1. Theoretical Analysis

#### 4.1.1. The Delivery Probability of Intra-Cluster Routing

According to the routing mechanism, nodes are divided into different clusters, and different forwarding methods are used for intra-cluster and inter-cluster routing. Assume that *T_N_* is the maximum delivery delay, then $P_{\text{intra}-\text{cluster}}^{ij}\left(\Delta t\leq T_{N}\right)$ is the delivery probability between any two nodes (for example, node *i* and node *j*) and Δ*t* is the time difference from the moment of the generation of a message until now. First, it is assumed that a message is delivered to node *j* in *T_M_*(*T_M_* ≤ *T_N_*), and node *k* in *T_M_* − 1, the probability $P_{\text{intra}-\text{cluster}}^{ij}\left(\Delta t=T_{M}\right)$ can be computed as:

(5) $$P_{\text{intra}-\text{cluster}}^{ij}\left(\Delta t=T_{M}\right)=\left(1-P_{\text{intra}-\text{cluster}}^{ik}\left(\Delta t=T_{M}-1\right)\right)\times {ɛ}_{kj}$$

$P_{\text{intra}-\text{cluster}}^{ij}\left(\Delta t\leq T_{N}\right)$ can be computed from the following relationship:

(6) $$P_{\text{intra}-\text{cluster}}^{ij}\left(\Delta t\leq T_{N}\right)=\sum_{1\leq T_{k}\leq T_{N}}P_{\text{intra}-\text{cluster}}^{ij}\left(\Delta t=T_{k}\right)$$

Since the contact probability between any two nodes in a same cluster should be greater than the threshold *γ*, to simplify the computation, we assume the contact probability is *γ* + Δ*γ*(0 ≤ Δ*γ, γ* + Δ*γ* ≤ 1) for any two nodes in the same cluster. We can get the minimum delivery probability $P_{\text{intra}-\text{cluster}}^{ij}\left(\Delta t\leq T_{N}\right)\prime$ as follows:

(7) $$\begin{array}{cc} P_{\text{intra}-\text{cluster}}^{ij}\left(\Delta t\leq T_{N}\right) & =\underset{1\leq k\leq T_{N}}{\text{∑}}P_{\text{intra}-\text{cluster}}^{ij}\left(\Delta t=k\right)\\ & =\left(\gamma +\Delta \gamma \right)+\left(1-\left(\gamma +\Delta \gamma \right)\right)\times \left(\gamma +\Delta \gamma \right)+\cdots +{\left(1-\left(\gamma +\Delta \gamma \right)\right)}^{T_{N}-1}\times \left(\gamma +\Delta \gamma \right)=1-{\left(1-\left(\gamma +\Delta \gamma \right)\right)}^{T_{N}}\geq 1-{\left(1-\gamma \right)}^{T_{N}}\\ & =P_{\text{intra}-\text{cluster}}^{ij}\left(\Delta t\leq T_{N}\right)\prime \end{array}$$

As a result, for the intra-cluster routing, when the threshold *γ* is determined, $P_{\text{intra}-\text{cluster}}^{ij}\left(\Delta t\leq T_{N}\right)\prime$ can be computed.

#### 4.1.2. The Delivery Probability of Inter-Cluster Routing

By the forwarding approach, when inter-cluster messages are generated, the messages will be forwarded to the relay nodes with a higher centricity until the messages are received by the destination or the nodes in the same cluster as the destination. In the transmission process, the messages will be carried by the relay nodes from one cluster to another, and finally be delivered to a relay node in the destination's cluster. Then, the intra-cluster forwarding scheme will be used to send the messages to their destinations.

As shown in the system model, the mobility model is a Markov process, where nodes will change from one state to another due to movement. For any node *k*, let *St_n_* is the *nth* state, and *P*(*s_i_, s_j_, t*) represents in the period time *t*, node *k* changes from the state *s_i_* to state *s_j_*. We can get the *P*(*s_i_, s_j_, t*) as:

(8) $$P(s_{i},s_{j},t)=P$$

In the Markov process, *P*(*s_i_, s_j_*) indicates the probability of node *k* from state *s_i_* to state *s_j_*. And *P*(*s_i_, s_j_*) can be represented as:

(9) $$P(s_{i},s_{j})=P(St_{n+1}=s_{j}|St_{n}=s_{i}$$

*G*(*s_i_, s_j_, t*) can be used to represent that the time from state *s_i_* to state *s_j_* is less than *t*. And *G*(*s_i_, s_j_, t*) is shown as:

(10) $$G\left(s_{i},s_{j},t\right)=P\left(\Delta t\leq t|St_{n+1}=s_{j},St_{n}=s_{i}\right)=\sum_{1\leq u\leq t}P\left(\Delta t=u|St_{n+1}=s_{j},St_{n}=s_{i}\right)$$

And then *P*(*s_i_, s_j_, t*) can be computed using *P*(*s_i_, s_j_*) and *G*(*s_i_, s_j_, t*) :

(11) $$\begin{array}{cc} P\left(s_{i},s_{j},t\right) & =P\left(St_{n+1}=s_{j},\Delta t\leq t|St_{n}=s_{i}\right)\\ & =P\left(\Delta t\leq t|St_{n+1}=s_{j},St_{n}=s_{i}\right)\times P\left(St_{n+1}=s_{j}|St_{n}=s_{i}\right)\\ & =G\left(s_{i},s_{j},t\right)\times P\left(s_{i},s_{j}\right) \end{array}$$

As *P*(*s_i_, s_j_, t*) is the probability of node *k* from state *s_i_* to state *s_j_* directly in the period time *t.* However, node *k* may enter another state and then enter the state *s_j_*, so we use *Q*(*s_i_, s_j_, t*) to represent this situation:

(12) $$\text{Q}\left(s_{i},s_{j},t\right)=\sum_{r=1}^{m}\sum_{u=1}^{t}\left(P\left(s_{i},s_{r},u\right)-P\left(s_{i},r,u-1\right)\right)\times Q\left(s_{r},s_{j},t-u\right)s_{j}\neq s_{i}$$

where *m* is the number of states that node *k* may enter. In other words, *Q*(*s_i_, s_j_, t*) is, in fact, the probability of node *k* from one cluster to another cluster *(P_cluster_*_−_*_cluster_*(Δ*t* ≤ *t*)). So we can get the inter-cluster delivery probability *P_inter_*_−_*_cluster_*(Δ*t* ≤ *t*) as:

(13) $$\begin{array}{ll} P_{\text{inter}-\text{cluster}}\left(\Delta t\leq T_{N}\right) & =\sum_{0\leq i<T_{N},0<j\leq T_{N},0\leq k<T_{N}}^{i+j+k\leq T_{N}}P_{\text{intra}-\text{cluster}}\left(\Delta t\leq i\right)\times P_{\text{cluster}-\text{cluster}}\left(\Delta t\leq j\right)\\ & \times P_{\text{intra}-\text{cluster}}\left(\Delta t\leq k\right) \end{array}$$

According to the Markov process, we can obtain the steady-state probability of node *i* as:

(14) $$P_{H}^{i}=\frac{{\left(P_{H}\right)}^{2}}{P_{H}+\left(1-P_{H}-P_{C}\right)\times \left(1-P_{H}\right)}$$

(15) $$P_{C}^{i}=\frac{P_{H}\times \left(1-P_{H}\right)}{P_{H}+\left(1-P_{H}-P_{C}\right)\times \left(1-P_{H}\right)}$$

(16) $$P_{\text{ELSE}}^{i}=\frac{\left(1-P_{H}-P_{C}\right)\times \left(1-P_{H}\right)}{P_{H}+\left(1-P_{H}-P_{C}\right)\times \left(1-P_{H}\right)}$$

where $P_{H}^{i}$ represents the steady-state probability of node *i* in *H*, $P_{C}^{i}$ represents the steady-state probability of node *i* in *C* and $P_{\text{ELSE}}^{i}$ represents the steady-state probability of node *i* in *H* which is not *I*'s home *H*. We can use these steady-state probabilities and the Markov process to approximately compute *P*(*s_i_, s_j_*) and *G*(*s_i_, s_j_, t*), and further *P_inter_*_−_*_cluster_*(Δ*t* ≤ *T_N_*) can be computed.

Since there is a limited capacity for the cache at each node, when a sufficient number of messages are generated, the cache could be overflowed, and the messages could be dropped. To compute the delivery probability, the service efficiency of the network *δ* should be known. Let *ρ* to be the ratio of customer service per unit time, we can obtain:

(17) $$\rho =\frac{1}{T_{N}}\sum_{i=1}^{T_{N}}P_{\text{inter}-\text{cluster}}\left(\Delta t\leq i\right)$$

Let *λ* to be the arrival rate of the messages, *B_Max_* to be the maximum cache available, we can compute the *δ* as:

(18) $$\delta =\left(\lambda *T_{N}*\rho +B_{Max}\right)/\left(\lambda *T_{N}\right)$$

Since we have obtained the delivery probability without considering the cache in formula (13), and according to the service efficiency, the delivery probability with the limited cache $P_{\text{inter}-\text{cluster}}^{\prime }\left(\Delta t\leq T_{N}\right)$ can be approximated as:

(19) $$P_{\text{inter}-\text{cluster}}^{\prime }\left(\Delta t\leq T_{N}\right)\approx \delta *P_{\text{inter}-\text{cluster}}\left(\Delta t\leq T_{N}\right)$$

#### 4.1.3. Average End-To-End Delay

As we all know, if the delivery ratio is close to 1, it indicates that most of the messages have been delivered. For intra-clustering routing, according to the formula (7), it is assumed *T_N_* = 9, the delivery ratio can be reached 1 − (1 − 0.4)^9^ = 0.99. That is to say, the messages could be delivered within maximum 9 time slots by the intra-clustering routing approach. Therefore, the average delay can be computed as:

(20) $$D_{\text{intra}-\text{clustering}}\approx \sum_{i=1}^{9}i*\left(\left(1-{\left(1-0.4\right)}^{i}\right)-\left(1-{\left(1-0.4\right)}^{i-1}\right)\right)=2.38$$

Like the intra-clustering routing, for inter-clustering routing, when the delivery ratio $P_{\text{inter}-\text{cluster}}^{\prime }\left(\Delta t\leq T_{N}\right)$ is close to 1, the average delay for inter-clustering routing can be obtained as:

(21) $$D_{\text{inter}-\text{clustering}}\approx \sum_{i=1}^{T_{N}}i*\left(P_{\text{inter}-\text{cluster}}^{\prime }\left(\Delta t\leq i\right)-P_{\text{inter}-\text{cluster}}^{\prime }\left(\Delta t\leq i-1\right)\right)$$

### 4.2. Complexity Analysis

**Figure 5.**
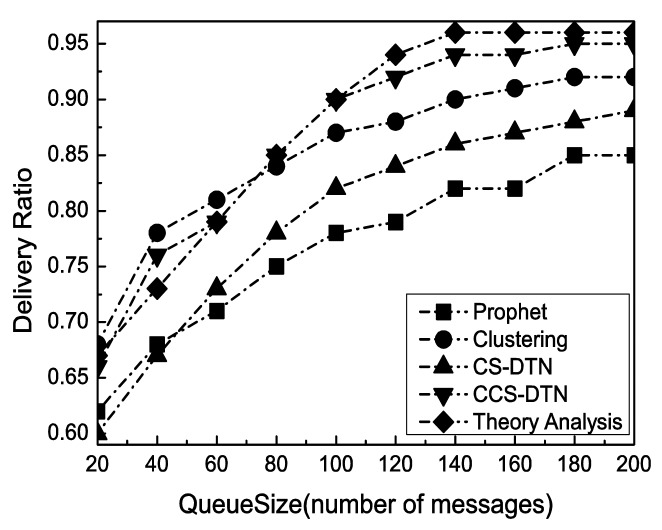
Impact of Queue Size on Delivery Ratio.

It is assumed that there are *M* nodes in the network, and from Figure 3, clustering in each time slot needs a maximum *M* comparisons for one node. That is to say, in each time slot there are maximum *M* × *M* comparisons, so the complexity of clustering operation is O(*M^2^*). On message forwarding, there are two scenarios to be considered. In the intra-clustering routing, since the Spray and Wait algorithm is adopted, there are limited numbers of moves for each message to be delivered and it needs only one comparison of contact probability for each move. Therefore the complexity of the intra-clustering routing is O(*N*) for *N* messages. On the other hand, in the inter-clustering routing, according to the algorithm in Figure 5, there are maximum of three comparisons for each move of a message. Like the intra-cluster routing, only limited number of moves are needed to deliver a message, so the complexity of the inter-clustering routing is O(*N*) for *N* messages. Finally, according to Definition 5, the algorithm needs O(*N^2^*) to perform network coding and decoding for *N* messages. Based on the above analysis, the complexity of the protocol at the worst case is O(*M^2^* + *N^2^*) for *M* nodes and *N* messages.

### 4.3. Simulation Analysis

The simulation has been performed by QUALNET. Simulation parameters are shown in Table 2.

In the simulation, we set P_H_ = 0.7, P_C_ = 0.1 for 90% of the nodes at each hot spot and $P_{H}^{\prime }=0.5$, $P_{C}^{\prime }=0.1$ for other 20% nodes. We compare the performance of our proposed solution with that of the Social Delay Tolerant Network based on Clustering (CS-DTN), Prophet [5] and Clustering [8]. The main difference between the CS-DTN and the CCS-DTN is that network coding has been used by the CCS-DTN scheme. Compared with the Prophet scheme, the CCS-DTN has adopted the clustering technology based on the community characteristics of the nodes in the social DTNs. The CCS-DTN has taken the advantages of the characteristics of the active nodes in the social DTNs while the Clustering schemes have not considered it. The successful delivery rate, defined as the ratio of the number of messages that have been successfully received by the destination over the number of messages that the source nodes have sent, and average end-to-end delay, defined as the average time used for a message transmitted from a source to its destination, have been measured. What's more, the simulation results have been compared with those obtained from the theory analysis.

**Figure 6.**
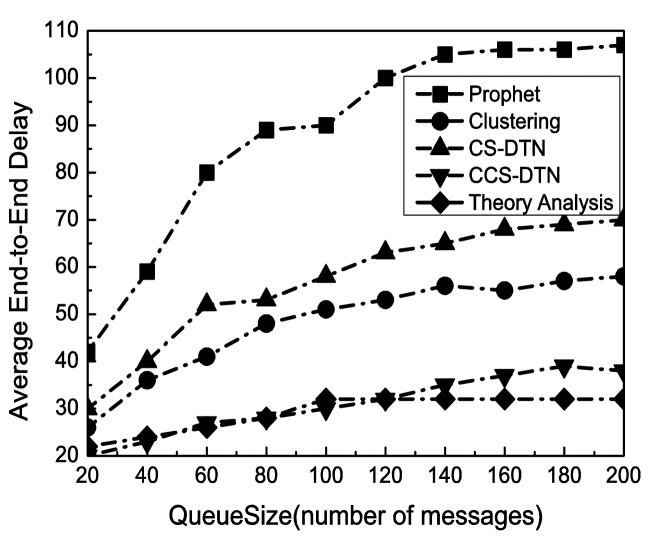
Impact of Queue Size on Average End-to-End Delay.

With the change of the buffer size at each node, the performance in terms of successful delivery ratio has been shown in Figure 5. Clearly, the successful delivery rate has been improved with the increase of the buffer size because the number of messages dropped due to overflows has been reduced. Meanwhile, as shown in Figure 6, as more messages have been kept in the buffer, the queuing delay has been increased. As a result, the average end-to-end delay has been increased. When the buffer size reaches to 140 messages, the delivery rate and average end-to-end delay become stable. It implies that the size of buffer is enough for the current states of the networks.

The Clustering scheme and the proposed CCS-DTN scheme have higher delivery rate than the Prophet scheme because they fully used the community characteristics of the nodes in social DTNs. Compared with the Clustering scheme, the CCS-DTN scheme has taken full use of the active characteristics of the nodes in the social DTNs and has introduced the network coding scheme. As a result, messages will be quickly delivered to the destination and some of dropped messages could be restored at the destination by the network coding scheme. Therefore, the CCS-DTN scheme can obtain a higher delivery rate than that of the Clustering scheme when the buffer is sufficient. On the contrary, when the buffer is small, the delivery rate of the CCS-DTN scheme is lower than that of the Clustering scheme. Since messages will be gradually transmitted to the nodes with a higher centrality by the CCS-DTN scheme, the nodes with a higher centrality will have to drop messages because there is no enough buffer. Besides, when the buffer is small, the delivery rate of the CCS-DTN scheme is also lower than the result of theoretical analysis. The reason is that in theoretical analysis, messages will be concentrated to the nodes with a higher centrality which leads to a buffer overflow at these nodes. But in actual simulation, messages may be delivered before they have been forwarded to the nodes with a higher centrality. In this way, there seem more messages dropped in theoretical analysis. On the other hand, we can find the improvements brought by the network coding technique by the comparison of the CCS-DTN scheme and the CS-DTN scheme. Using the network coding technology, some of dropped messages have been restored at the destination. What's more, the average end-to-end delay of the CCS-DTN scheme is lower than those of other protocols. By the Clustering scheme, routing discovery has to be performed when messages are forwarded between clusters. While by the CS-DTN scheme, there are more retransmissions than that of the CCS-DTN scheme because of the use of network coding technology.

**Figure 7.**
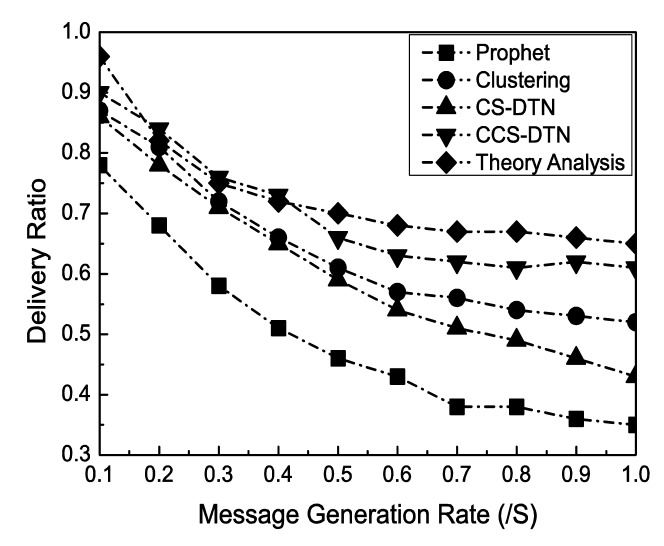
Impact of Message Generation Rate (/s).

From Figure 7, it is clear that the delivery rate declines when the message generation rate becomes higher. The reason is that the nodes have to drop messages as more and more messages are generated in the network and each node has a limited buffer size. But, the change of the delivery rate by the CCS-DTN scheme is lower than those by others schemes. One reason is that by the CCS-DTN scheme messages will be quickly delivered to the destination. Another reason is that by using of the load balancing mechanism, the CCS-DTN scheme can utilize the buffer more efficiently. And the network coding technique has also played a part in improving the delivery rate. Moreover, it is found that the results obtained from the mathematical analysis and the results from simulations have been proved each other to show the consistency.

**Figure 8.**
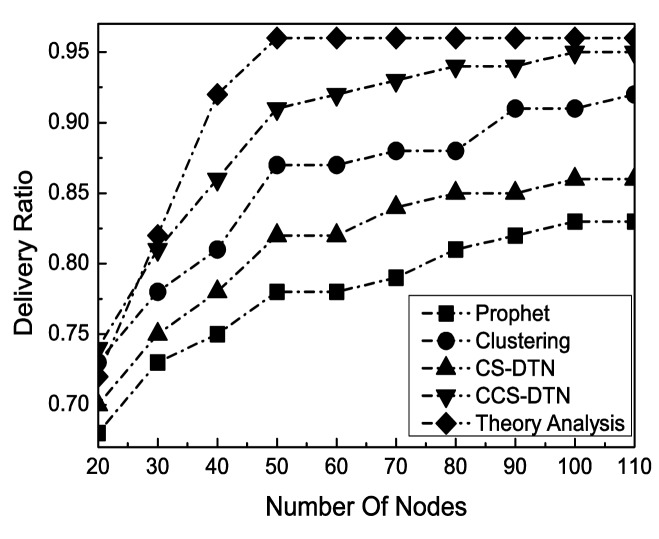
Impact of Number of Nodes on Delivery Ratio.

Another change on the network states for the evaluation is the number of nodes. As shown in Figure 8, the average delivery ratio increases with the increase of the number of the nodes. When there are more nodes in the network, the total available buffer in the network will be larger. And there are more selectable relay nodes which can increase the probability of success delivery. However, when the number of the nodes exceeds a certain threshold, it could lead to bottlenecks at some nodes. As a result, the delivery ratio will be stable in a range.

**Figure 9.**
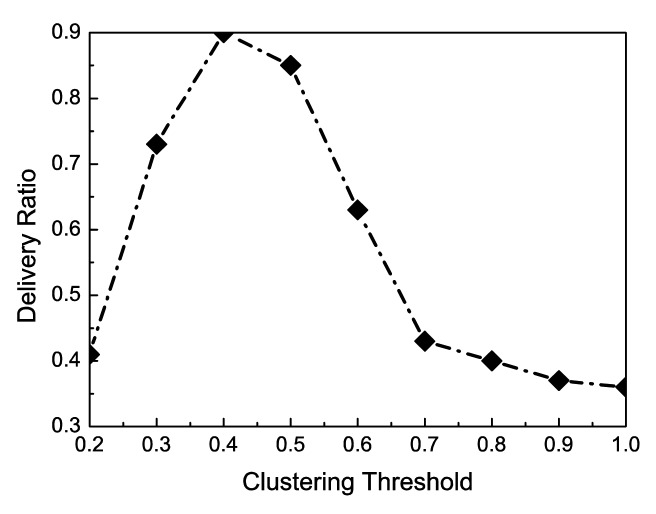
Impact of the Threshold of Contact Probabilty for Clultering on Delivery Ratio.

The last simulation is to study the impact of the clustering threshold on the delivery ratio. As mentioned in Section 3, after clustering, the contact probability between any two nodes in a same cluster must be greater than a value which is defined as the clustering threshold. From Figure 9, it is clear that the delivery ratio first increases with the increase of the clustering threshold and then declines when the threshold exceeds a certain value. When the threshold is very small, all nodes will be in one cluster. The delivery ratio is very low. The reason is that there are only limited copies of a message and the contact probability between the source node and the destination node is low. On the other hand, when the threshold is large, all nodes will be grouped into different clusters, some of which may contain only one or two nodes.. By the inter-cluster routing of the CCS-DTN scheme, messages will be gradually transmitted to the nodes with a higher centrality. In this way, many messages will be dropped when there is not enough buffer in the nodes with a higher centrality. Therefore, when the threshold exceeds a certain value, the delivery ratio cannot be high.

According to Equation (14), the probability of one node, node *i*, in its *H* is $P_{H}^{i}=0.645$. Assume that any two nodes, node *i* and node *j*, are in the same *H*. The probability when they are in *H* at the same time is $P_{H}^{i}*P_{H}^{j}=0.645*0.645=0.416$. That is to say, 0.4 is a right value to be set as the clutering threshold. According to Figure 9, it is obvious that when the clustering threshold is 0.4, the delivery ratio can achieve the highest value. Therefore, the results from the theoretical analysis and those from the simulation are consistent.

Based on the above analysis, in general, the proposed CCS-DTN scheme can obtain a better performance in the scenarios under study. Meanwhile, it is clear that the cache is a bottleneck for the CCS-DTN scheme to achieve a higher performance.

## 5. Conclusions

Taking into account of the characteristics of DTN nodes in social scenarios and the advantage of network coding, we have proposed a CCS-DTN routing algorithm, by which mobile nodes will be divided into different clusters according to the contact probability of each mobile node. The Spray and Wait algorithm is used to perform the intra-cluster routing, while the social characteristics, the centrality of nodes and the network coding technique have been employed to perform the inter-cluster routing. The analytical evaluation and simulation results show that in the social scenarios, a better performance in terms of a higher delivery ratio and a lower average end-to-end delay could be achieved by the CCS-DTN scheme. Simulation results have also been proven to be consistent with the theoretical analysis.

## Figures and Tables

**Table 1.** List of Notations.

**Symbol**	**Quantity**
*ε_ij_*	Contact Probability between node *i* and *j*
*α*	A const value between 0 and 1
*γ*	Clustering threshold
*C_i_*	The cluster which includes node *i*
*S_i_*	The stability of node *i* in *C_i_*
*M_i_*	Members in *C_i_* which are recorded by node *i*
$T_{i}^{j}$	Contact time between node *i* and *j*

**Table 2.** Simulation Parameters.

**Parameter**	**Value**
Simulation Time	10,000 s
Size of Area	3000 × 3000
Warm-up Time	500 s
Number of Nodes	50, 20–110
Traffic	CBR
Number of Messages for Coding	20
Transmission Range	250 m
Message Size	150 Byte
Number of Message Copies	2
Threshold of Clustering	0.4
Rate of Message Sending	0.1
MAC Protocol	802.11
